# Retreatment With Flow Diverters and Coiling for Recurrent Aneurysms After Initial Endovascular Treatment: A Propensity Score-Matched Comparative Analysis

**DOI:** 10.3389/fneur.2021.625652

**Published:** 2021-06-03

**Authors:** Wenqiang Li, Wei Zhu, Xinguo Sun, Jian Liu, Yang Wang, Kun Wang, Ying Zhang, Xinjian Yang, Yisen Zhang

**Affiliations:** ^1^Department of Neurosurgery, The First Affiliated Hospital of Zhengzhou University, Zhengzhou, China; ^2^Department of Interventional Neuroradiology, Beijing Neurosurgical Institute and Beijing Tiantan Hospital, Capital Medical University, Beijing, China; ^3^Department of Neurosurgery, Binzhou People's Hospital, Binzhou, China; ^4^Department of Neurosurgery, Beijing Chaoyang Hospital, Capital Medical University, Beijing, China

**Keywords:** recurrent intracranial aneurysms, endovascular re-treatment, flow diverters, parent artery stenosis, recanalization

## Abstract

**Background:** Flow diverters and conventional coiling are established modalities for the retreatment of intracranial recurrent aneurysms after initial endovascular treatment. We aimed to compare the efficacy of these techniques.

**Methods:** We retrospectively analyzed data for patients with recurrent aneurysms after initial endovascular treatment retreated in our center with either a pipeline embolization device (PED) or conventional coil embolization from January 2012 to July 2020. We performed 1:2 propensity score matching (PSM) using the nearest neighbor method. We controlled for: initial treatment strategy, aneurysm size, neck diameter, symptom presentation, history of aneurysm rupture, age, sex, fusiform-dissecting aneurysm, bifurcation aneurysm, and aneurysm location. The clinical and morphological factors of all patients at initial treatment and the angiographic and clinical results at the second treatment were collected and compared between the propensity-matched pairs.

**Results:** A total of 105 intracranial aneurysms were identified; 18 patients (17.1%) were treated with a PED, and 87 (82.9%) were treated via conventional coil embolization. PSM resulted in 12 matched pairs (12 patients in the PED group and 24 in the coiling group). There was no significant difference of ischemic and hemorrhagic complications between the groups, the obliteration rate of branches covered by stent, or modified Rankin Scale scores at the last clinical follow-up. Importantly, the retreatment strategy in the PED group provided significantly different results vs. the coiling group (*P* < 0.001), with a lower recurrence rate (0.0 vs. 29.2%, respectively; *P* = 0.037). However, the procedural failure rate and the parent artery stenosis were more frequently in PED group compared with coiling group (both were 16.7 vs. 0.0%; *P* = 0.040).

**Conclusions:** Endovascular retreatment for recurrent aneurysms after initial endovascular treatment might be safe and effective. Flow diverters might be associated with reduced risk of recanalization and an increased risk of procedural failure and mild parent artery stenosis.

## Introduction

Coil embolization is a well-established treatment modality to prevent the rupture of intracranial aneurysms; however, the method has a higher technique failure rate and recurrence rate in complex aneurysms (1–5). With advances in endovascular technology, flow diverters have become an increasingly established treatment in the management of complex intracranial aneurysms. Compared with other endovascular treatment, the higher aneurysm occlusion rate and lower aneurysm recurrence rates was found (6–9). Recurrent aneurysm carries a persistent risk of regrowth and rupture, and retreatment is considered necessary to avoid bleeding or rebleeding (10–12). However, choice of treatment modality differs between institutions. Previous studies reported encouraging results with endovascular treatment (coil embolization with or without stents and flow diverters) for recurrent aneurysms (6, 13–17). However, comparative data between flow diverters and conventional coil embolization in recurrent aneurysms are extremely limited. Moreover, non-randomized studies may have been affected by selection bias, and a propensity score analysis could address this bias. In this study, using propensity score matching, we compared the safety and efficacy of retreatment in patients with recurrent aneurysms after initial endovascular treatment who were retreated with either a pipeline embolization device (PED; Covidien, Boulder, CO) or coil embolization.

## Materials and Methods

### Patient Selection

This is a retrospective, matched, case–control study, which was approved by the ethics committee of our hospital. We reviewed the medical records and image data in our aneurysm database, which included patients diagnosed with intracranial aneurysms between January 2012 and July 2020. All patients included in this study met the following inclusion criteria: (1) intracranial aneurysms confirmed by digital subtraction angiography and treated with endovascular treatment; (2) recanalization at the first follow-up angiography and re-treated with endovascular treatment; and (3) further follow-up angiographic imaging to determine whether the aneurysm had recanalized after retreatment. All patients who underwent retreatment of the recurrent aneurysm with PED were identified; 18 patients received a PED, and 87 patients underwent coil embolization. For each case receiving a PED, we matched two controls from the patients undergoing coil embolization, using propensity score matching to reduce imbalances in the baseline characteristics. Ultimately, 12 patients with recurrent aneurysms were re-treated with PEDs and 24 patients undergoing conventional coiling treatment were included after propensity score matching. We defined patients receiving a PED as the PED group and patients undergoing coil embolization as the coiling group. Clinical and morphological factors for all included patients at initial treatment were collected and analyzed, namely age, sex, history of subarachnoid hemorrhage (SAH), symptomatic presentation at initial treatment, smoking and drinking history, hypertension, diabetes mellitus, hyperlipidemia, aneurysm size, aneurysm neck, aneurysm location, bifurcation aneurysm, and treatment strategy. Data for the modified Rankin scale (mRS) score before treatment, at discharge, and at follow-up; treatment strategy; stent number; stent extension; ischemic and hemorrhage complications; immediate and follow-up angiographic results; results of the parent artery and branches covered by the stent; and the follow-up period to recurrent aneurysm at re-treatment were also collected.

### Interventional Procedures

All patients received standard dual antiplatelet therapy (100 mg aspirin and 75 mg clopidogrel) for 3–5 days before the procedure if stent protection was anticipated in patients with unruptured aneurysms. Loading doses of 300 mg clopidogrel and 300 mg aspirin 4 h before treatment were administered in patients with ruptured aneurysms. Platelet function testing (thromboelastography and genotype of CYP2C19) was assessed to identify hyporesponders, and the antiplatelet regimen was adjusted in these patients. All procedures were performed under general anesthesia, and full procedural heparinization was used to achieve a targeted activated clotting time of 250–300 s. For the coil embolization, properly-shaped microcatheters were introduced over microguidewires and navigated into the recurrent aneurysm cavity under the guidance of the microguidewire. Aneurysms were packed as densely as possible with coils. Stent-assisted coiling was used if coils were unstable in the aneurysm or if coil herniation occurred. The jailing technique was the usual method, in which a stent was deployed after the microcatheter was in position, and the first coil was deployed but not detached. For aneurysms with a prior stent, mesh technology was used to deliver the coils to the aneurysm. Following the procedure, dual antiplatelet therapy was continued for at least 1 month, and aspirin was continued for 6 months, thereafter.

For patients receiving a PED, we adopted a triaxial support system to access the aneurysm. The PED was introduced through a Marksman microcatheter (Covidien), delivered to the parent artery defect, and then deployed. Several endovascular techniques (namely the use of wires, catheters, or balloon angioplasty) were performed if the device was inadequately expanded. For aneurysms treated with a PED and coiling, similar techniques were performed, such as stent-assisted coiling. Patients continued taking dual antiplatelet therapy (300 mg aspirin and 75 mg clopidogrel) for 3 months after the procedure, at which time, clopidogrel was discontinued. We used aspirin monotherapy thereafter, indefinitely.

### Clinical and Angiographic Evaluation

All procedural technical and clinical complications were documented. Procedural failure was defined as incomplete expansion of the stent spanning the entire stented segment of the parent artery (1). Ischemic complications constituted thromboembolic events associated with re-treatment, namely ischemic stroke, transient ischemic attack, stent thrombosis, urgent revascularization, and new ischemic lesions with evidence in magnetic resonance imaging. Hemorrhagic complications were confirmed on CT images. Clinical outcomes were assessed using mRS score, and scores of 0–2 were considered favorable outcomes; mRS scores > 2 were considered poor outcomes.

Immediate and follow-up angiographic outcomes were graded using the Raymond classification, which included complete occlusion, neck remnant, and sac remnant (18). The first angiographic follow-up was scheduled 3–6 months post-operatively. The follow-up angiographic outcomes were classified into three categories, and changes were compared with the immediate results in the same projections to assess (1) improvement, defined as decreased contrast filling in the aneurysmal sac; (2) stable, defined as unchanged contrast filling in the aneurysmal sac; and (3) recurrence, defined as increased contrast filling in the aneurysmal sac. Patency of the branches covered by the stent and in-stent stenosis were also documented. The outcomes were evaluated by two neurointerventionalists with 5 years of experience in endovascular treatment who evaluated blinded images. Disagreements were resolved by a third neurointerventionalist with 10 years of experience in endovascular treatment.

### Statistical Analysis

We performed matched case–control analysis using propensity score matching. The underlying characteristics considered in the propensity score estimation was the initial treatment strategy, aneurysm size, neck diameter, symptom presentation, history of SAH, age, gender, fusiform-dissecting aneurysm, bifurcation aneurysm, and aneurysm location. Categorical variables are reported as proportions, and continuous variables are presented as the mean ± standard deviation. We assessed the balance between the PED group and the coiling group using Wilcoxon's rank-sum test for non-parametric variables, and compared categorical variables using the Chi-squared test. Statistical analyses were performed using statistical software (SPSS, version 21.0; IBM Corp., Armonk, NY). The level of statistical significance was set at *P* < 0.05.

## Results

### Patients' Demographics and Aneurysm Characteristics

A total of 105 intracranial aneurysms qualified for the study; 18 (17.1%) patients were treated with a PED, and 87 patients (82.9%) were treated via coil embolization (Table 1). Significant differences between the PED group and the coiling group were observed. Specifically, aneurysm size and neck diameter in the PED group were larger (13.30 ± 6.40 vs. 10.35 ± 6.92, *P* = 0.046 and 8.65 ± 4.63 vs. 6.44 ± 3.79, *P* = 0.035); patients in the PED group were more likely to be asymptomatic at presentation (50.0 vs. 23.0%; *P* = 0.009), and to have bifurcation aneurysm (77.8 vs. 49.4%, *P* = 0.028) compared with patients in the coiling group, respectively. While the patients with previous history of subarachnoid hemorrhage were less in PED group (22.2 vs. 52.9%, *P* = 0.018). Moreover, the characteristic, namely age, between the two groups had significant trends (*P* < 0.1).

**Table 1:** Characteristics of patients and aneurysms at initial endovascular treatment before and after propensity score matching.

	**Before propensity score matching**			**After propensity score matching**		
	**PED (*n =* 18)**	**Coiling (*n =* 87)**	***P-*value**	**PED (*n =* 12)**	**Coiling (*n =* 24)**	***P-*value**
Age	48.60 ± 14.17	42.77 ± 12.65	0.084	51.08 ± 11.95	47.71 ± 9.11	0.391
Female, %	8 (44.4)	56 (64.4)	0.115	7 (58.3)	14 (33.3)	1.000
History of SAH, %	4 (22.2)	46 (52.9)	0.018^*^	3 (25.0)	12 (50.0)	0.151
Presentation			0.009^*^			0.373
Asymptomatic, %	9 (50.0)	20 (23.0)		4 (33.3)	5 (20.8)	
Headache, %	5 (27.8)	51 (58.6)		5 (41.7)	15 (62.5)	
Oculomotor paralysis, %	3 (16.7)	16 (18.4)		2 (16.7)	4 (16.7)	
Weakness of limbs, %	1 (5.6)	0 (0.0)		1 (8.3)	0 (0.0)	
**Risk factors**						
Smoking, %	4 (22.2)	20 (23.0)	0.944	3 (25.0%)	5 (20.8)	0.777
Drinking, %	2 (11.1)	16 (18.4)	0.456	1 (8.3)	4 (16.7)	0.496
HTN, %	6 (33.3)	31 (35.6)	0.853	3 (25.0)	9 (37.5)	0.453
DM, %	1 (5.6)	9 (10.3)	0.529	0 (0.0)	2 (8.3)	0.303
HLD, %	1 (5.6)	6 (6.9)	0.836	1 (8.3)	1 (4.2)	0.607
Aneurysm size	13.30 ± 6.40	10.35 ± 6.92	0.046^*^	14.82 ± 4.67	16.17 ± 7.09	0.827
Neck diameter	8.65 ± 4.63	6.44 ± 3.79	0.035^*^	8.37 ± 4.36	8.89 ± 5.37	0.920
Fusiform-dissecting, %	5 (27.8)	12 (13.8)	0.143	3 (25.0)	4 (16.7)	0.551
Aneurysm location			0.866			0.905
ICA, %	12 (66.7)	61 (70.1)		8 (66.7)	16 (66.7)	
ACA/AcomA, %	2 (11.1)	13 (14.9)		1 (8.3)	2 (8.3)	
MCA, %	1 (5.6)	4 (4.6)		0 (0.0)	1 (4.2)	
Posterior, %	3 (16.7)	9 (10.3)		3 (25.0)	5 (20.8)	
Bifurcation aneurysm, %	14 (77.8)	43 (49.4)	0.028^*^	9 (75.0)	14 (58.3)	0.326
Initial treatment strategy			0.112			1.000
Stent alone, %	2 (11.1)	2 (2.3)		1 (8.3)	2 (8.3)	
Coils + stent, %	12 (66.7)	51 (58.6)		9 (75.0)	18 (75.0)	
Coiling, %	4 (22.2)	34 (39.1)		2 (16.7)	4 (16.7)	

*SAH, subarachnoid hemorrhage; HTN, hypertension; DM, diabetes mellitus; HLD, hyperlipidemia; mRS, modified Rankin score; ACA, anterior cerebral artery; AcomA, anterior communicating artery; ICA, internal carotid artery; MCA, middle cerebral artery*.

* *p < 0.05*.

### Propensity Score-Adjusted Characteristics

Following propensity score adjustment and 1:2 matching, 12 aneurysms treated with PEDs and 24 aneurysms treated with conventional coil embolization were matched. The patients in the PED group and coiling group were matched on the basis of similarities in their demographic and aneurysm characteristics, and all covariates were statistically indistinguishable between the two groups (Table 1).

### Propensity Score-Adjusted Outcomes of Retreatment

In the PED group, eight patients (66.7%) were retreated using a PED alone, and four (33.3%) were treated with a combination of a PED and coils. Significant difference was found between the PED group and coiling group (15 patients with stent-assisted coiling and 9 with coiling without a stent) (*P* < 0.001). A single pipeline stent was used in 11 patients (91.7%) and two stents were used in 1 patient (8.3%). All patients in the coiling group received a single stent (100%). Two patients (16.7%) in the PED group experienced procedural failure because of inadequate PED expansion, and required balloon angioplasty. These patients received a self-expanding stent at the initial treatment. Treatment was successful in the patients in the coiling group. There is significant difference of procedural failure rate between two groups (8.3 vs. 0.0%, *P* = 0.04).

#### Angiographic Outcomes

**Figure 1:**
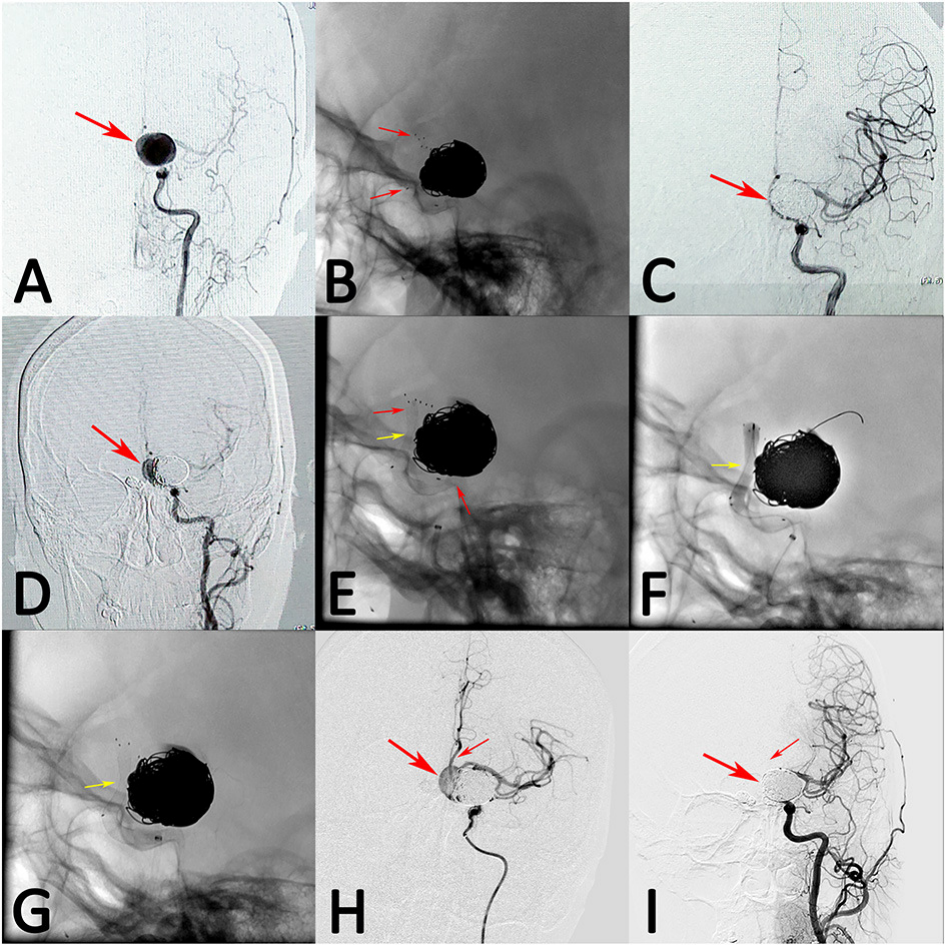
A patient with left giant posterior communicating artery aneurysm was treated with stent-assisted coil embolization (**A**, arrow). The stent deployed successfully and the aneurysm was occluded completely after treatment (**B,C**, arrows). However, the aneurysm recanalized at 3 months follow-up (**D**, arrow). The recanalized aneurysm was retreated with single pipeline embolization device, while the device was inadequately expanded (**E**, middle arrow). Balloon angioplasty were performed, while the device was still expanded inadequately (**F**,**G**, arrow). However, the blood flow of aneurysm was stasis and the distal blood flow was sufficient after treatment (**H**, arrows). At 12 months follow-up, angiograph result showed that the aneurysm had complete occlusion, while the anterior cerebral artery was obliteration (**I**, arrows).

Treatment, complications, angiographic and follow-up results were showed in Table 2. In the immediate post-operative angiographic images, PED treatment resulted in complete aneurysm occlusion in 6 (50.0%) patients, neck remnant in 4 (33.3%) patients, and aneurysmal sac remnant in 2 (16.7%) patients. Five visible branches were covered by the PED. In the coiling group, 6 (25.0%) patients had complete aneurysm occlusion, 17 (70.8%) had neck remnants, 1 (4.2%) had an aneurysmal sac remnant, and six visible branches were covered by the stent. Angiographic follow-up data were available for all patients at a median of 8.33 months in the PED group. One patient died following hemorrhagic complications after PED treatment. Another thirteen aneurysms were stable or improved. Two patients (16.7%) developed asymptomatic mild in-stent stenosis (<25.0%) and were managed conservatively; both patients had previously undergone stent-assisted coil embolization. One patient with giant aneurysm had complete occlusion after PED retreatment, while the anterior cerebral artery was obliteration at follow up (Figure 1). However, the patient had no ischemic symptoms at last follow up.

**Table 2:** Treatment, complications, angiographic, and follow-up results of the recurrent aneurysm after 1:2 matching by propensity score.

	**PED group (*n =* 12)**	**Coiling group (*n =* 24)**	***P-*value**
Pre-procedure mRS			0.414
≤ 2, %	10 (83.3)	17 (70.8)	
>2, %	2 (16.7)	7 (29.2)	
Treatment strategy			<0.001^*^
Stent alone, %	8 (66.7)	0 (0.0)	
Stent + coiling, %	4 (33.3)	15 (62.5)	
Coiling, %	0 (0.0)	9 (37.5)	
Number of stents			0.151
1, %	11 (91.7)	24 (100.0)	
>1, %	1 (8.3)	0 (0.0)	
Procedural failure, %	2 (16.7)	0 (0.0)	0.040^*^
Ischemic complications, %	1 (8.3)	1 (4.2)	0.607
Hemorrhage complications, %	1 (8.3)	0 (0.0)	0.151
mRS at discharge			0.201
≤ 2, %	10 (83.3)	23 (95.8)	
>2, %	2 (16.7)	1 (4.2)	
Follow-up mRS			0.151
≤ 2, %	11 (91.7)	24 (100.0)	
>2, %	1 (8.3)	0 (0.0)	
Mean follow-up duration (months)	8.33 ± 4.29	10.75 ± 9.21	0.736
Immediate angiographic results			0.085
Complete occlusion, %	6 (50.0)	6 (25.0)	
Neck remnant, %	4 (33.3)	17 (70.8)	
Sac remnant, %	2 (16.7)	1 (4.2)	
Follow-up angiographic results			0.037^*^
Improved or stable, %	12 (100.0)	17 (70.8)	
Recurrence, %	0 (0.0)	7 (29.2)	
Parent artery			0.040^*^
Patency, %	10 (83.3)	24 (100.0)	
Stenosis, %	2 (16.7)	0 (0.0)	
Branches covered by stent
Patency at follow-up	4/5 (80.0)	6/6 (100.0)	0.251

*FD, flow diverter; mRS, modified Rankin score*.

* *P < 0.05*.

**Figure 2:**
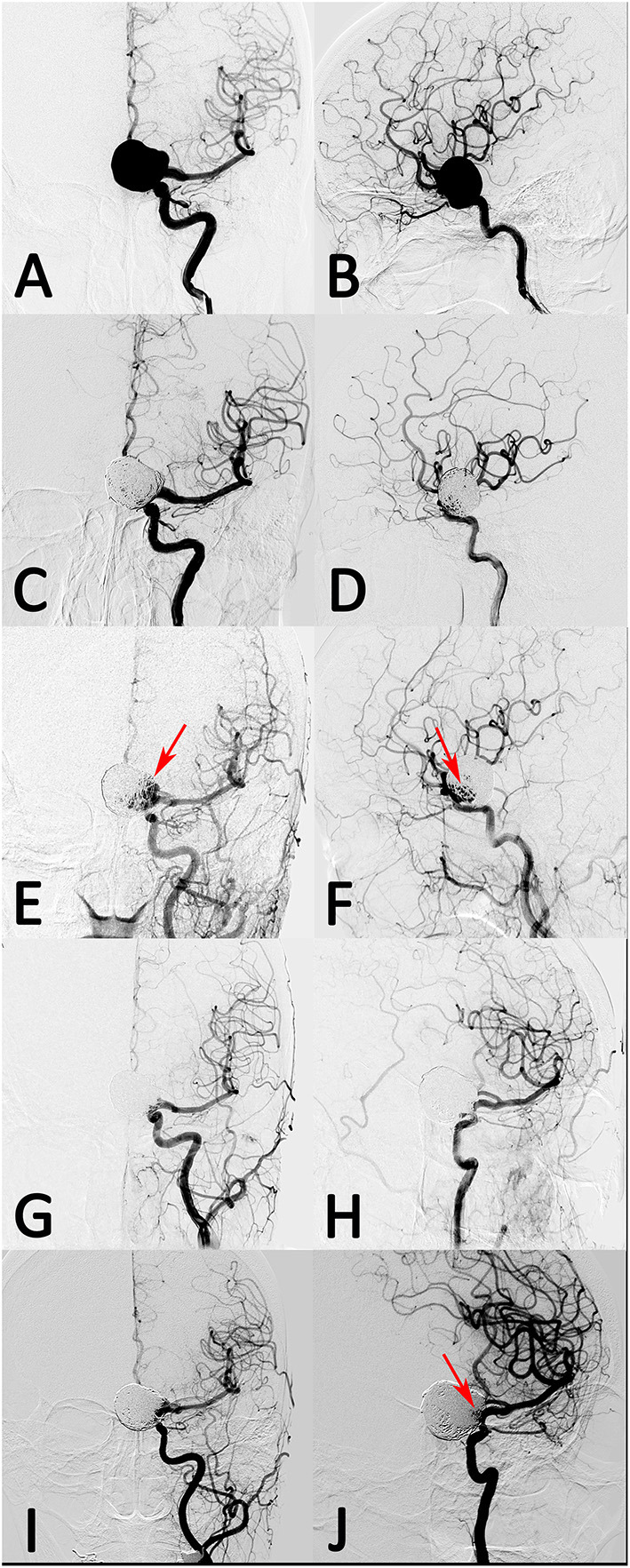
A patient with left giant posterior communicating artery aneurysm was treated with stent-assisted coil embolization. **(A,B)** The aneurysm was occluded near-completely after treatment. **(C,D)** However, the aneurysm recanalized at 10 months follow-up. (**E,F**, arrow) The recanalized aneurysm was retreated with stent-assisted coil embolization and had complete occlusion after treatment. **(G,H)** However, the aneurysm had re-recanalization at 8 months follow-up angiograph result (**I,J**, arrow).

In the coiling group, angiographic follow-up data were available for all patients at a median of 10.75 months, and seven patients (29.2%) developed aneurysm recurrence (three with initial coiling and four with initial stent-assisted coiling). The parent artery of all patients was patent. A case example with giant aneurysm showed in Figure 2 had recurrence after coiling retreatment.

The recurrence rate in the PED group (0.0%) was lower compared with the coiling group (29.2%), and the difference was statistically significant (*P* = 0.037). However, the parent artery treated with the PEDs more frequently achieved stenosis (PED group vs. coiling group: 16.7 vs. 0.0%, respectively; *P* = 0.040). The obliteration rate of branches covered by the stents between PED and coiling group at the follow-up angiography had no significant difference (*P* = 0.251). Fifteen patients were treated stent-assisted coiling and 9 with coiling alone. In the 15 patients with stent placement, 5 patients treated with LVIS stent, 9 with Enterprise stent, and 1 with Solitaire stent. There is no significant difference in complications, angiographic and follow-up results between different stents (Supplementary Table 1).

#### Complications and Clinical Outcomes

In the PED group, two (16.7%) patients had complications, constituting one ischemic complication and one hemorrhagic complication. The ischemic patient, who had a giant right cavernous aneurysm, was retreated with a single PED, and experienced left hemiparesis. The hemorrhagic patient with a right vertebral artery aneurysm previously treated with stent-assisted coiling developed a brainstem hemorrhage after PED treatment and died (mRS = 6). Intraprocedural angiography showed that the guide wire had perforated a basilar artery. One patient retreated with stent-assisted coiling in the coiling group developed a minor ischemic complication appearing as left-sided hemiparesis and blurry vision in the right eye after treatment. At the latest clinical follow-up, the two patients with ischemic complications had almost recovered, with minimal residual hemiparesis or mild blurry vision, respectively (mRS <2). There was no difference in the mRS scores before retreatment, at discharge, and at the latest follow-up between the PED and coiling groups. Eleven of the 12 (91.7%) patients (PED group) and all patients (100%) (coiling group) experienced overall favorable outcomes (mRS: 0–2) at the latest follow-up, without developing new neurological deficits.

## Discussion

### Key Results

Endovascular treatment has been expected to induce the progressive occlusion of recurrent aneurysms; however, outcomes among the various endovascular treatment modalities cannot be easily or accurately compared. The baseline characteristics are significantly different between the PED and coiling groups, which we showed in this study. We balanced these characteristics using propensity score matching, and the results demonstrated that the PED group had significantly lower recanalization rates during follow-up after retreatment of recurrent aneurysms compared with the coiling group, with a concomitant higher procedure failure and mild parent artery stenosis rate. Furthermore, there was no significant difference in ischemic and hemorrhagic complications between the two groups.

### Current Modalities in the Retreatment of Recurrent Aneurysms

Endovascular embolization is an accepted and preferred technique for the treatment of intracranial aneurysms. However, a major issue encountered with endovascular treatment is the rate of aneurysm recanalization. Approximately 37.5–90% of all cerebral aneurysms undergoing reconstructive endovascular treatment recur, and recanalization rates remain high with stent-assisted coiling, ranging from ~20 to 57% (19). Complex aneurysms, such as large and giant intracranial aneurysms (≥ 10 mm), have a higher annual rupture rate compared with small aneurysms, and require treatment (20). However, such complex intracranial aneurysms were initially considered unamenable to conventional endovascular coil embolization. Moreover, several risk factors (namely, packing density ratio, prior rupture status, large aneurysm size, young age, and incomplete initial occlusion) predict aneurysm recurrence in conventional endovascular treatment (21). Surgical retreatment for previously coiled aneurysms with or without adjunctive stenting is challenging. If manipulated the stented artery directly or extruded the previous coil mass, it might tear the parent artery and increase the risk of thromboembolism. Therefore, endovascular treatment has been widely accepted as a treatment modality for recurrent aneurysms with previous endovascular treatment (11). However, conventional endovascular retreatment still had the risk of repeat recurrences, for which most treated aneurysms are vulnerable, with recurrent risk factors (13). The advent of flow diverters brings a new endovascular tool for reconstructive treatment and vascular remodeling for these complex aneurysms, and these devices provide a high complete aneurysm occlusion rate. However, limited information is available regarding the use of flow diverters as treatment for recurrent intracranial aneurysms after prior coil embolization, and the effectiveness and safety of flow diverters in the retreatment of recurrent aneurysm is unclear compared with conventional coiling retreatment.

### Conventional Coiling in the Retreatment of Recurrent Aneurysms

Although additional coiled treatment of aneurysms with coiling previously is associated with a low procedural complication rate and sufficient occlusion in most aneurysms, the risk of complications is also increased in the retreatment of recurrent aneurysms. Ringer et al. (22) aimed to evaluate the risks of retreatment for recurrent or residual aneurysms by endovascular coiling, they found that permanent major disability occurred in patients with multiple treatment-related deaths more than the case with once. However, in our study, favorable clinical outcomes were achieved in all patients in the coiling group; only one patient with stent-assisted coiling suffered ischemic complications, and the patient recovered with a favorable clinical outcome at the latest follow-up.

Re-embolization of a previously treated intracranial aneurysm is often considerably more technically challenging than the initial treatment. Re-embolization with coils can be used if the recurrent cavity is large enough and has an appropriate morphology. The microcatheter tip should be carefully positioned in the center of the recurrent cavity; however, confirming this placement may be difficult. Furthermore, re-embolizing aneurysms using coils alone was associated with a higher re-recanalization rate. Previous studies reported that the re-recanalization rates in re-embolized aneurysms were higher than the previously reported recanalization rates after initial coil embolization, with the recanalization rate of retreated aneurysms ranging from ~44.1 to 48.6% (23–25). With these therapeutic limitations, stent-assisted techniques might be an alternative treatment to better protect the coils in the aneurysm and reduce the rates of further recanalization. Cho et al. (25) and Jeon et al. (26) reported that stent usage could stabilize the inserted coils, preserve parent artery patency, induce a persistent flow diversion effect, and confer a protective effect to prevent further recanalization. However, 16% of the studies' patients required additional endovascular treatment because of coil compaction and regrowth of a recurrent aneurysm after stent-assisted embolization of recurrent aneurysms. Furthermore, the risks associated with stent-assisted retreatment were markedly higher than in previous reports of aneurysm re-embolization (27). We found similar results in our study, with a higher recurrence rate with retreatment with coiling compared with flow diverters, while the aneurysmal recurrence rate after stent-assisted coiling was lower than that with coils alone. Moreover, the recurrent aneurysms in our study after matching were large or giant aneurysms with higher recanalization rates than rates reported in previous studies.

### Flow Diverters in the Retreatment of Recurrent Aneurysms

Flow diverters are a new treatment strategy for intracranial aneurysms. The flow diverter had a strong hemodynamic effect to reduce the flow of aneurysm, further gradually occludes with thrombus organized and neointima formation (9). Flow diverter treatment has a lower probability of recurrence than conventional coiling, even in lesions vulnerable to recurrence. Therefore, using flow diverters as a novel technique should be attempted to improve the outcomes of endovascular retreatment in recurrent aneurysms. Endovascular treatment with PEDs for recurrent aneurysms with previous coiling alone is a safe, effective, and durable treatment option (14). However, in previous studies, flow diverter treatment after stent placement was reportedly less effective and more complicated, with 40.9–75.0% occlusion rates and a higher complication rate of up to 16.7% (1, 13, 28–30). Several technical issues regarding flow diverter deployment within a previously placed stent might be the key factors, including incomplete opening of the flow diverter, interrupted visibility of the flow diverter, and anchoring of the flow diverter with a drag-and-drop technique (13). In our study, technique-related issues occurred in three patients with previous stent implantation, and incomplete extension of the PED occurred in two patients and resulted in mild stenosis of the parent artery. The procedural failure rate in PED group was significantly higher than that with conventional coiling. However, the patients with parent artery stenosis had no symptoms during follow-up; patients were managed conservatively, and had favorable clinical outcomes. However, another patient died from brain stem hemorrhage because of basilar artery perforation.

Equivalent efficacy with the use of flow diverters in the treatment of recurrent aneurysms has been demonstrated in numerous studies (6, 14, 15, 28–32). Yu et al. (33) reported a 0% recanalization rate for previously treated aneurysms after PED placement, and Kühn et al. (15) reported that the complete or near-complete occlusion rate ranged from 83.3 to 100% at follow-up, confirming the efficacy of flow diverters in previously coiled aneurysms. These data suggest that aneurysms recurring after previous treatment may be effectively managed with a PED. In our study, no aneurysms recanalized during follow-up, and the recanalization rate of aneurysms treated with a flow diverter was significantly lower than that with conventional coiling. Furthermore, the ischemic and hemorrhagic complication rates did not differ significantly between patients treated with a PED vs. coiling. The present study demonstrated better angiographic findings and good clinical outcomes in patients treated with a PED compared with patients treated with conventional coiling, and the results in patients with PEDs were better than results in previous studies. These improved outcomes might be due to rapid developments of treatment devices and more gaining experience of operators in PED treatment.

### Limitations

The major limitation of this study is its retrospective, single-center design. Although propensity score-based matching analysis was used as a statistical approximation to reduce patient selection bias, this statistical technique still has limitations, namely noticeable shrinkage of the cohort's sample size. Furthermore, the shorter duration of follow-up in the PED cohort compared with the coiling group represents another significant limitation. Studies with long-term angiographic follow-up are needed.

## Conclusion

Endovascular retreatment for recurrent aneurysms after initial endovascular treatment was safe and effective. Flow diverters might be beneficial for reducing the risk of recanalization in recurrent intracranial aneurysms, with an increased risk of procedural failure and mild parent artery stenosis, which might be caused by incomplete extension of the flow diverter.

## Data Availability Statement

The raw data supporting the conclusions of this article will be made available by the authors, without undue reservation.

## Ethics Statement

The studies involving human participants were reviewed and approved by the ethics committee of Beijing Tiantan Hospital. Written informed consent for participation was not required for this study in accordance with the national legislation and the institutional requirements.

## Author Contributions

WL performed the statistical analysis and the manuscript writing. WL, WZ, XS, JL, and KW acquired the data. WL, YisZ, YinZ, and YW contributed to data analysis and interpretation. YisZ and XY contributed to the experimental design and manuscript revision, handled funding, and supervision. All authors contributed to the article and approved the submitted version.

## Conflict of Interest

The authors declare that the research was conducted in the absence of any commercial or financial relationships that could be construed as a potential conflict of interest.
