# Supplementary material for: Retreatment With Flow Diverters and Coiling for Recurrent Aneurysms After Initial Endovascular Treatment: A Propensity Score-Matched Comparative Analysis
Source: Front Neurol. 2021 Jun 3;12:625652. doi: 10.3389/fneur.2021.625652 (PMC8209336; doi:10.3389/fneur.2021.625652)
Supplement: Supplementary file 1 [file Table_1.docx]

**Supplemental Table 1** Complications, angiographic and follow-up results of the recurrent aneurysm with stent placement in coiling group after 1:2 matching by propensity score.

|  | Lvis stent (n=5) | Enterprise stent (n=9) | Solitaire stent (n=1) | P value |
| --- | --- | --- | --- | --- |
| Ischemic complications, % | 0 (0.0) | 0 (0.0) | 0 (0.0) | 1.000 |
| Hemorrhage complications, % | 0 (0.0) | 0 (0.0) | 0 (0.0) | 1.000 |
| Immediate angiographic results |  |  |  | 0.600 |
| Complete occlusion, % | 2 (40.0) | 3 (33.3) | 0 (0.0) |  |
| Neck remnant, % | 3 (60.0) | 6 (66.7) | 1 (100.0) |  |
| Sac remnant, % | 0 (0.0) | 0 (0.0) | 0 (0.0) |  |
| Follow-up angiographic results |  |  |  | 0.385 |
| Improved or stable, % | 4 (80.0) | 8 (88.9) | 1 (100.0) |  |
| Recurrence, % | 1 (20.0) | 1 (11.1) | 0 (0.0) |  |
